# Effects of cooling on pig heart excitation and contraction

**DOI:** 10.3389/fcvm.2026.1753083

**Published:** 2026-03-09

**Authors:** Mei Li, Linus B. Persson, Matthias Schwartzkopf, Erik Steen, Ann Terry, Björn Wohlfart, Stig Steen, Anders Arner

**Affiliations:** 1 Department of Clinical Sciences, Lund University, Lund, Sweden; 2 Department of Physics, Lund University, Lund, Sweden; 3 Deutsches Elektronen-Synchrotron (DESY), Hamburg, Germany; 4 Department of Automatic Control, Lund University, Lund, Sweden; 5 MAX IV Laboratory, Lund University, Lund, Sweden

**Keywords:** electrophysiology, hypothermia, Langendorff, skinned fibers, x-ray diffraction

## Abstract

Although variations in temperature have a profound impact on cardiac function, little is known regarding the excitation and contractile parameters over a broad temperature interval. In view of the clinical implications of lowered temperature in resuscitation and in cardiac preservation/evaluation for transplantation, we have examined the contractile function using Langendorff perfused hearts and isolated trabecular muscle from pig, in combination with electrophysiology and x-ray diffraction. Lowered temperature in the range 37°C–22°C was associated with an increase in systolic pressure and active force. In permeabilized preparations, force and Ca^2+^ sensitivity decreased with temperature, showing that the increased force down to 22°C in the intact heart and trabeculae was not due to changes in thin filament regulation, but most likely to increased activator [Ca^2+^]. At lower temperature (<22°C), force of the heart decreased, suggesting that the temperature effects in the regulatory system became dominating. ECG analysis showed that frequency was lowered and that PQ-, QS- and QT- times were prolonged at lower temperature. This was associated with a gradual depolarization of the cell membrane, prolonged action potential and an attenuation of the fast upstroke phase. These changes in rise time and amplitude of the action potential would predispose for uneven propagation and arrhythmia as temperature is lowered. At the same time, the prolonged action potential can be associated with an increased [Ca^2+^] at lower temperature. Small angle x-ray diffraction showed that the filament lattice of intact trabecular muscle tended to swell at low temperature (10°C vs. 22°C) and revealed a mass transfer from myosin to actin filaments, which would reflect changes in cellular physiology and contractile system structure at low temperature.

## 1 Introduction

The human heart is normally working at the physiological temperature around 37°C, and maintenance of core body temperature is a key homeostatic mechanism. Still, cardiac temperature can be altered in some important clinical situations. One example is exposure to cold environment that can result in accidental hypothermia, defined as core temperatures below 35°C. In the USA, about 1,500 persons die annually from accidental hypothermia (1, 2). Temperatures below about 28°C increase the risk for cardiac arrhythmias and cardiac arrest which are key causes for death in severe hypothermia. The detrimental effects can be partly counteracted by effects of slowed cell metabolism at low temperature. Indeed, people have survived after long exposure to severe hypothermia below 15°C–20°C with circulatory arrest (3–5). Cold temperature can be beneficial in some clinical scenarios, and therapeutic cooling around 30°C has been used clinically e.g., as organ protection, in brain surgery and in cardiac surgery (6, 7). Ice arrest of the heart has even been reported to be used in open cardiac surgery (8). In the field of cardiac transplantation, cold cardioplegia (around 4°C–10°C) is a standard procedure for donor heart preservation prior to transplantation (9).

Research in our laboratory using pig hearts has shown that the heart can be preserved, with maintained coronary and myocardial function, for up to 24 h using non-ischemic conditions with high-K^+^ cardioplegia at 8°C (10, 11). This technique has also been applied in transplantation of human hearts (12). Our current interest is to evaluate the donor heart *ex vivo* prior to transplantation, to ensure optimal cardiac function (13). In view of these aspects and the issues with accidental hypothermia mentioned above, we noted that very little is known regarding the effects of varied temperature on cardiac performance, in particular regarding the effects on the hearts from larger animals. The aim of this study was therefore to examine the effects of varied temperature in a broader interval using beating perfused whole hearts in a Langendorff preparation and isolated intact and permeabilized trabecular preparations, in combination with electrophysiology and x-ray scattering analysis of the contractile system. The experiments were performed using hearts from pigs, which are similar in function to the human hearts and of current interest due to the prospect of using pig hearts in xenotransplantation (14–18).

## 2 Methods

### 2.1 Animals and heart isolation

Swedish domestic pigs of both genders (males castrated after birth) weighing 40–50 kg (age 90–120 days) were used. The animals were handled in compliance with the European Convention for the Protection of Vertebrate Animals Used for Experimental and Other Scientific Purposes (Directive 2010/63.EU). The experiments were approved by the local Animal Ethical Committee (5.8.18-15906/2020). The pigs were anesthetized with an intramuscular injection of atropine 0.5 mg (Unimedic AB, Matfors, Sweden), xylazine 100 mg (Bayer, Solna, Sweden), and ketamine 20 mg/kg body weight (Intervet AB, Stockholm, Sweden). Thereafter an intravenous injection of fentanyl 4 µg/kg (Braun, Melsungen, Germany) and midazolam 0.4 mg/kg (Hameln Pharma Plus GmbH) was given. Anesthesia was maintained with an intravenous infusion of ketamine (10 mg/(kg × h)) and rocuronium bromide (1.5 mg/(kg × h), Fresenius Kabi Austria GmbH, Graz, Austria) via an ear vein. Tracheostomy was performed and the pigs were connected to a respirator with volume-controlled and pressure-regulated ventilation. Sternotomy was done and the right atrium was cannulated for collection of whole blood. The distal ascending aorta was clamped and cold cardioplegic solution (Plegisol® solution) was then administered through a needle inserted into the aorta proximal to the clamp. When the heart was completely relaxed it was removed and kept in cold cardioplegic solution for 30 min before use.

### 2.2 Langendorff preparations

**Figure 1:**
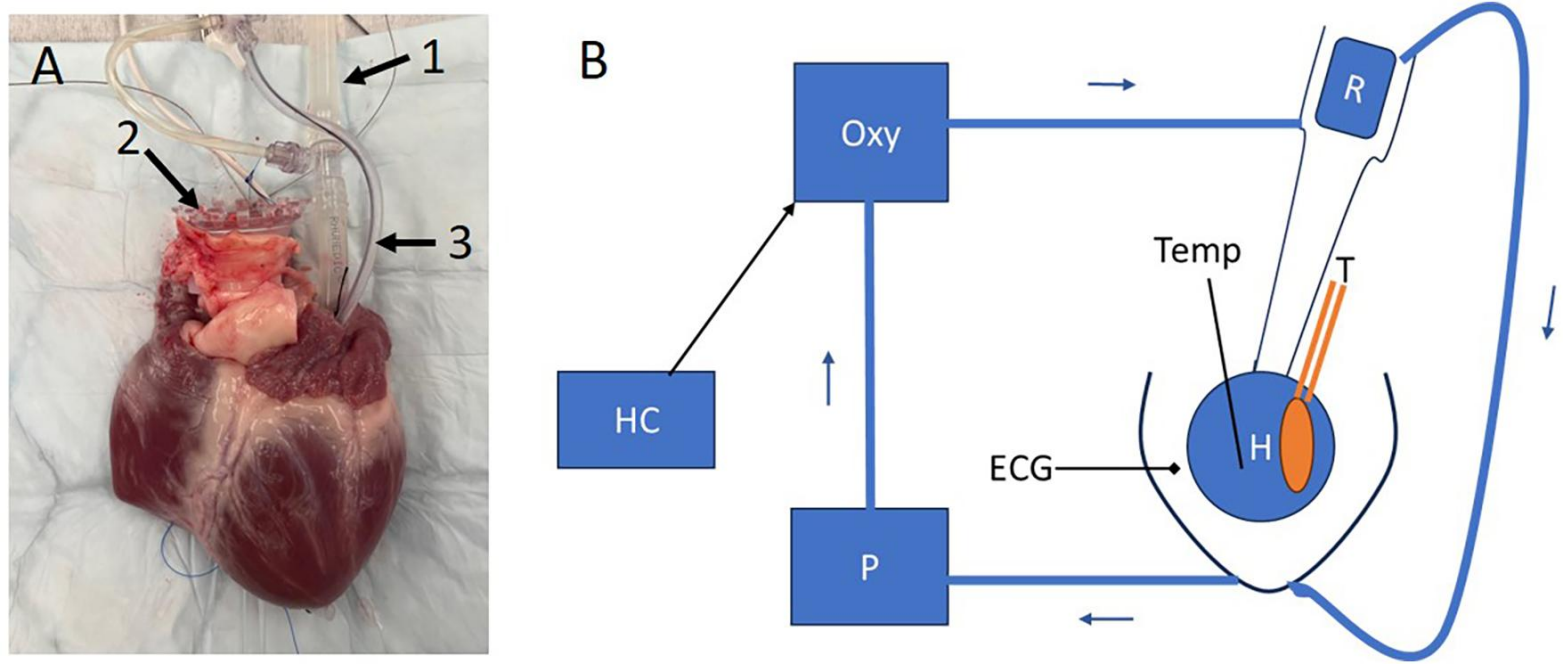
The Langendorff set-up for perfusing the heart. **(A)** Photograph of a heart with balloon inserted in the left ventricle via the left atrium (arrow 1). Arrow 2 indicates the attachment between aorta and perfusion apparatus. Arrow 3 is the strangulator for tightening the purse string suture fixing the balloon to the mitral valve opening. A temperature probe was inserted in the right ventricle. **(B)** Shows schematically the system, where the heart (H) was submerged in the blood containing solution. The balloon was attached to a pressure transducer (T). A Stöckert roller pump (P) pumped the blood from the bath through an oxygenator (Oxy) into the aorta. The temperature was controlled via a heater/cooler (HC) and continuously monitored in the heart. A resistance (R) determined the perfusion pressure. The excess perfusion, not entering the coronary system was directed back to the bath. ECG electrodes were placed in the fluid close to the heart.

A rubber latex balloon was attached to a 3D-printed plastic holder and inserted into the left ventricle. The tip of the balloon was tied with silk thread and fixed via a small opening in the apex of the heart to prevent the balloon from being forced out during beating. The other end of the balloon was fixed with a purse string suture in the mitral annulus. The pressure in the balloon itself was close to 0 mmHg when filled to 40 ml. Figure 1A, shows the heart with a balloon inserted in the left ventricle. The setup is schematically illustrated in Figure 1B. The balloon was filled with physiological saline, and attached to a pressure transducer (T in Figure 1B). A temperature probe was inserted in the right ventricle and ECG was monitored via surface electrodes. The perfusion solution (total volume about 2 L) was made of a mixture of whole blood and dextran-40 (100 g/L) in physiological saline (0.5 L per 2 L perfusion solution) giving a hematocrit around 25% (13, 19). The aortic pressure was controlled and maintained above 40 mmHg, to enable closure of the aortic valves, via a resistance (R, Figure 1B) in the aortic inlet and by the speed of the pump (P, Figure 1B). On average the aortic pressure was 70 ± 6 mmHg (*N* = 5). This gave a coronary flow of about 0.5–1 L/min. Throughout the experiment, blood gases, glucose and electrolytes were measured at regular intervals and adjusted when required. All hearts received a slow infusion (0.5 ml/h) of a cocktail containing per 50 ml: adrenaline (1 mg), noradrenaline (1 mg), hydrocortisone (100 mg), cocaine (1 mg) and triiodothyronine (0.3 mg), cf (10, 20). When the heart was mounted in the system, heated to 37°C, and exhibiting regular beating, the balloon was filled with saline to a volume (30–40 ml) giving maximal developed pressure during the heart beats. In some cases, the heart developed ventricular fibrillation initially, which was corrected by defibrillation. Ethanol (0.3%) was added to prevent ventricular fibrillation during cooling (21).

When stable contractions were obtained at 37°C, the temperature was changed using the heater-cooler (HC, Figure 1) in steps: 37–30, 22, 15 and 10°C as measured in the right ventricle and followed by return to 37°C. Recordings of balloon pressure and ECG were made about 15 min at each temperature after systolic pressure had stabilized. The amplitude, the rise and fall rates of the pressure transients were evaluated together with some ECG parameters (heart rate, PQ-, QS- and QT-time).

### 2.3 Isometric force recordings from isolated trabeculae

Hearts were isolated as described above and thin, non-branching, trabecular muscle strips (diameter 1–2 mm, length about 5 mm) were carefully isolated from the right ventricular inner wall. Using silk thread (6/0) the preparations were mounted between a fixed pin and a Grass FT03 force transducer in temperature controlled open glass baths containing Krebs’ solution (in mM): NaCl 123, NaHCO_3_ 20, KH_2_PO_4_ 1.2, KCl 4.7, MgCl_2_ 1.2, CaCl_2_ 1.5, glucose 5.5. The solution was gassed with 95% O_2%_ and 5% CO_2_ giving a of pH 7.4 at 37°C. The samples were stimulated via platinum wires placed alongside the muscles, using 0.5 ms pulses, 0.2 Hz at supramaximal voltage from a Grass S48 stimulator (Grass, Quincy. Mass. USA) and a current amplifier. The force responses were recorded using an A/D converter and the Chart 4 program (ADInstruments, Oxford, UK). After 30 min accommodation the muscle length was adjusted to optimal for active force (approximately 1.3 × slack length). This degree of stretch gives about 2.3 µm sarcomere length (22). Temperature of the solution was changed in steps: 37°C, 30°C, 22°C, 15°C and 10°C. The muscles were exposed to each temperature for 5 min (enabling the force to stabilize) and we evaluated the twitch responses (amplitude, rates of contraction and relaxation) as well as the passive tone.

Since CO_2_ solubility is affected by temperature, cooling of the Krebs’ solution results in a small drop in pH (by 0.22 pH units from 37°C to 10°C). To examine if the responses to temperature was due to pH changes, we also performed temperature experiments in MOPS buffered solution (3-(N-Morpholino)propanesulfonic acid, MOPS 20, NaCl 118, KCl 5, Na_2_HPO_4_ 1.2, MgC_2_ 1.2, CaCl_2_ 1.6, glucose 10 mM, pH 7.4), which has a stable pH over a range of temperature gassed with air. This solution did not alter force responses at 37°C compared to those in Krebs’ solution, and gave a similar temperature dependence. To explore if changes in resting force were dependent on active contraction, we also examined temperature effects on tone in preparations in the presence of the muscle relaxant 2,3-butanedione monoxime, BDM (50 mM).

### 2.4 Membrane potentials

Trabecular preparations were mounted in a temperature-controlled Perspex bath between a fixed pin and a Kistler-Morse force transducer. The samples were held in Krebs’ solution and stimulated via a stimulus isolation unit either with a bipolar electrode at one end of the muscle or with field stimulation using platinum electrodes on each side of the preparation. Stimulation frequency was 0.2 Hz, duration 0.2 ms and supramaximal voltage was applied. The muscles were stretched to optimal length. Membrane potentials were recorded at different temperatures (37°C, 30°C, 22°C, 15°C and 10°C) using KCl filled (3M) glass micro electrodes (resistance about 10–20 MΩ) as described previously (22). Membrane potential (resting potential, action potential shape and duration) as well as twitch responses (amplitude, rates of contraction and relaxation) were recorded.

### 2.5 Force and Ca^2+^-sensitivity in permeabilized preparations

Muscle bundles from the left ventricle were permeabilized and stored as described previously (23, 24). Thin strips (diameter about 200 µm, length 1–2 mm) were teased out, and mounted using cellulose glue between a micrometer screw for length adjustment and an AE801 force transducer (Kronex, Oakland, USA) in 0.5 ml temperature-controlled Perspex baths. The samples were stretched to about 1.3 × slack length [corresponding to 2.3 µm sarcomere length (22)]. After 30 min in relaxing {pCa=-log_10_[(Ca^2+^)] 9} solution with 1% Triton X-100 (for additional permeabilization) and 5 min in relaxing solution at 22°C, each preparation was activated at pCa 4.7 at 22°C to determine a control force response. The muscle was then relaxed (pCa 9) and examined at one of different temperatures (37°C, 30°C, 22°C, 15°C and 10°C). The active tension was determined at increasing Ca^2+^ levels (pCa 9.0, 6.6, 6.0, 5.7, 5.4 and 4.7). The force at each Ca^2+^ level was normalized to the initial control response at 22°C. The force and [Ca^2+^] data of each sample was analyzed by fitting a hyperbolic function to determine the *EC_50_* (i.e., the concentration giving half-maximal tension), the maximal response (in relation to control response) and the residual tension at pCa 9 after the final contraction. The solutions contained 20 mM MOPS buffer, 0.5 mM Mg^2+^ (adjusted with MgAcetate), 5 mM MgATP, ionic strength 200 mM (adjusted with K-proprionate), 6 mM ethylene glycol-bis (β-aminoethyl ether)-*N*,*N*,*N*′,*N*′-tetraacetic acid, EGTA (in pCa 9 solution and CaEGTA in pCa 4.7 solution), 10 mM phosphocreatine, PCr, 2 mM dithioerythritol, DTE and 320 U/mL creatine kinase. The composition of the solutions was calculated as described (25) with binding constants corrected for temperature and ionic strength (26). The apparent Ca^2+^ binding constants for EGTA at the different temperatures were [37°C: 6.467; 30°C: 6.442; 22°C: 6.414; 15°C: 6.389; 10°C: 6.365 log (M^−1^)]. In one series of experiments, we also examined the responses at 37°C using solutions with higher MgATP (10 mM) and phosphocreatine (20 mM). Rigor solutions contain zero MgATP, phosphocreatine and creatine kinase.

### 2.6 Small angle x-ray diffraction

Small angle x-ray scattering (SAXS) was used to examine the effects of temperature on myofilament organization. The experiments were performed at the CoSAXS beamline, MAX IV synchrotron light facility, Lund, Sweden and at the P03 beamline, Petra III, DESY, Hamburg (27), as described previously (24, 28). Intact and permeabilized trabecular preparations were isolated as described above and mounted horizontally using silk thread in a temperature-controlled cuvette equipped with Kapton windows. Intact preparations were held in the MOPS buffered physiological solution and skinned fibers in solutions described above (section 2.3 and 2.5). The solution for intact muscle was gassed with air and exchanged every 5–10 min. The x-ray beams (wavelength 1 Å CoSAXS and 0.95 Å at P03), had sizes at the sample of about 50 × 60 µm (CoSAXS) and 16 × 22 μm (P03). The sample detector distance was set to 3.5 m (CoSAXS) and 5.1 m (P03) which gave a good resolution of the equatorial pattern using exposures of 0.5–1 s. Rat tail collagen was used for calibration. The samples were moved between exposures to prevent beam damage. Scattering patterns were recorded at CoSAXS using an EIGER 2 X 4M detector (Dectris AG, Baden-Daetwil, Switzerland) and at P03 with a PILATUS 300K detector (DECTRIS Ltd.). A polynomial function was fitted to the equatorial intensity signal for background subtraction and the center of mass for the reflections was determined. The preparations were stretched to optimal length (1.3 × slack length) and exposed to different temperatures. We evaluated the spacing of the 1.1 and 1.0 reflections, and using integration of the peak intensities, the 1.1/1.0 intensity ratio (24).

### 2.7 Statistics

Results are expressed as mean ± standard error of the mean (SEM). Statistical comparisons and curve fitting (least-squares) were performed using routines implemented in SigmaPlot14 (Alfasoft AB, Gothenburg, Sweden) or GraphPad Prism (GraphPad Software, Boston, USA). Student's *t*-test was used when comparing two groups and analysis of variance (ANOVA) when several groups were compared. For values not normally distributed, a non-parametric test (Mann–Whitney rank sum test) was used. *N* is the number of preparations or hearts.

## 3 Results

### 3.1 Recordings from langendorff perfused hearts

After mounting at 37°C, a regular stable beating was established *in vitro* as the cardioplegia solution was washed out and replaced by the blood-containing oxygenated physiological solution. The balloon volume was gradually increased to a volume (∼40 ml) where the developed pressure was maximal. Preliminary experiments showed that when temperature was lowered to 22°C and below, ventricular fibrillation occurred in a few heart preparations. This was prevented by adding a low amount of ethanol to the perfusion solution. We did not observe any major changes (<5%) in ECG parameters (PQ, QS, QT times) or pressure responses when ethanol was administered at 37°C. Since ethanol has been shown to prevent cold induced arrhythmia (21), we routinely added 0.3% ethanol to the solution and no events with ventricular fibrillation were observed in the five experiments included.

**Figure 2:**
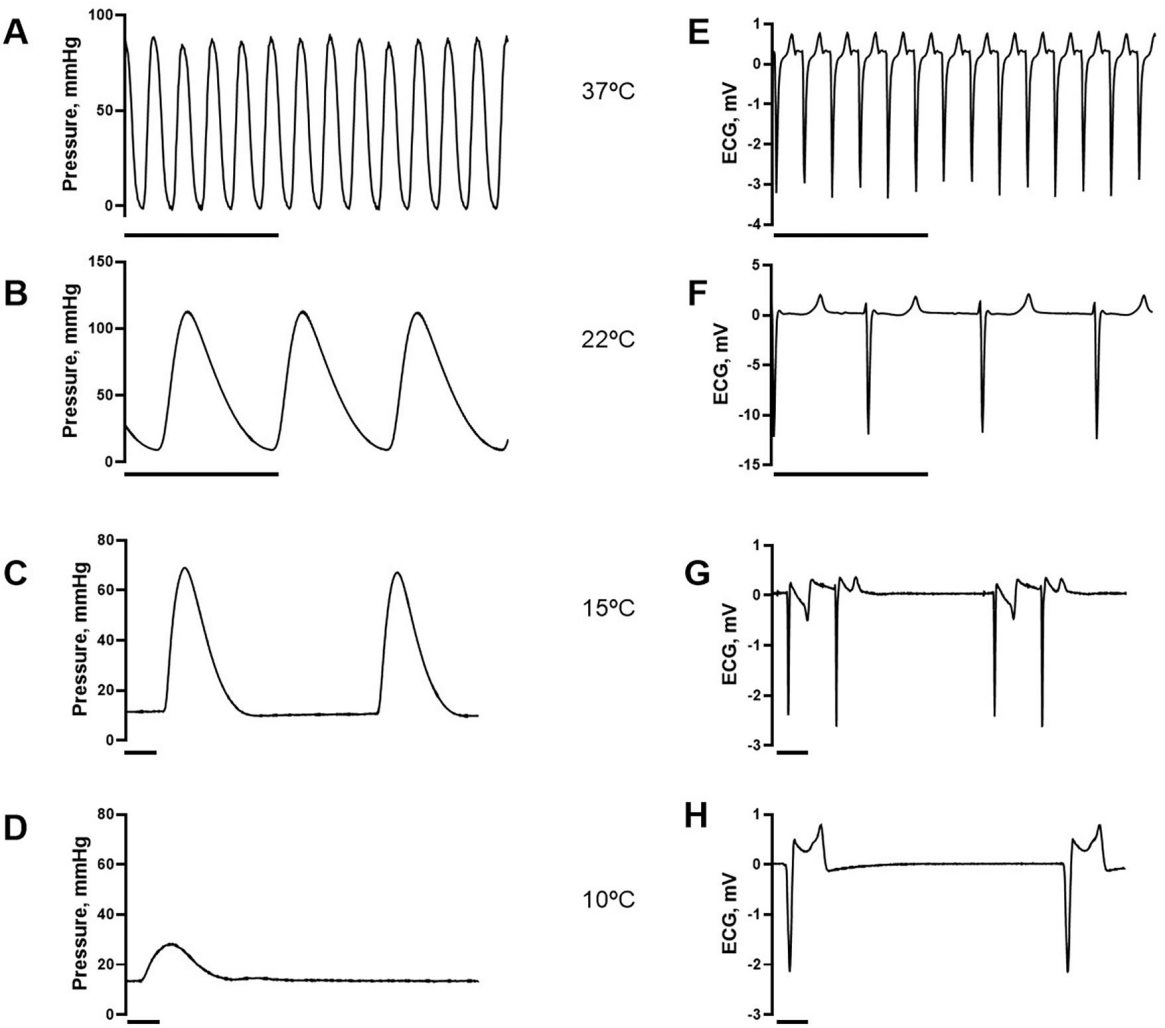
Original recordings of balloon pressure **(left A–D)** and ECG **(right E–H)** at different temperatures (**A, E**: 37°C; **B, F**: 22°C; **C, G**: 15°C; **D, H**: 10°C). Horizontal lines indicate 2 s.

**Figure 3:**
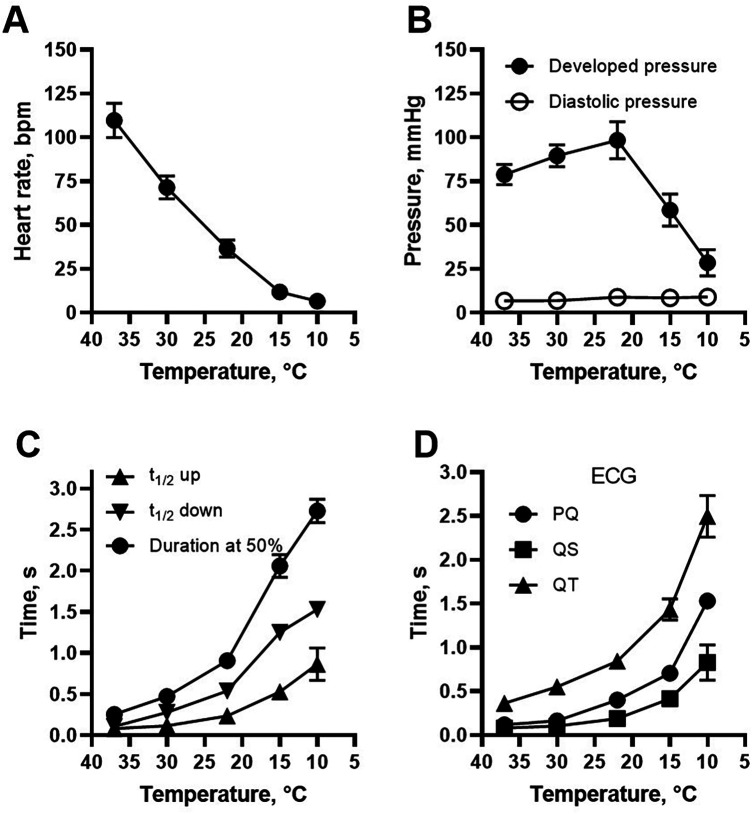
Contractile and ECG parameters of the perfused heart at different temperatures. **(A)** Shows heart rate, **(B)** shows developed (systolic) and diastolic pressure. **(C)** Shows half-times of contraction and relaxation, and duration of the twitch at half amplitude of the heart beats. The ECG parameters, PQ-, QS- and QT-time are summarized in **(D)** *N* = 5, except for PQ time *n* = 4 at 15°C and *n* = 1 at 10°C.

Figure 2 shows the recorded changes in contractile parameters and ECG from the isolated beating heart when temperature was lowered. Figure 3 shows the summarized data. Most prominently, the heart rate was significantly reduced with cooling and was at 10°C less than 1/10 of that at 37°C (Figure 3A). The developed pressure increased gradually by about 20% from 37°C to 22°C, and then dropped at lower temperatures (Figure 3B). The low diastolic pressure recorded in the balloon was increased by a small, non-significant, amount at lower temperature. The half-time of contraction and relaxation were increased with cooling. At 10°C, the half-times were about 10 times longer compared to those at 37°C (Figure 3C). At lower temperatures, the ECG measurements showed that the time intervals (PQ, QS and QT times) were gradually increased (Figure 3D). At 15°C and below, sinus arrhythmias and AV block usually occurred (cf. Figures 2G,H). A complete AV block was observed in 1 preparation at 15°C and in 4 of 5 at 10°C. When temperature was increased again to 37°C, the developed pressure and rates of contraction and relaxation recovered to more than 80% of their initial values.

### 3.2 *In vitro* recordings of isolated trabecular preparations

**Figure 4:**
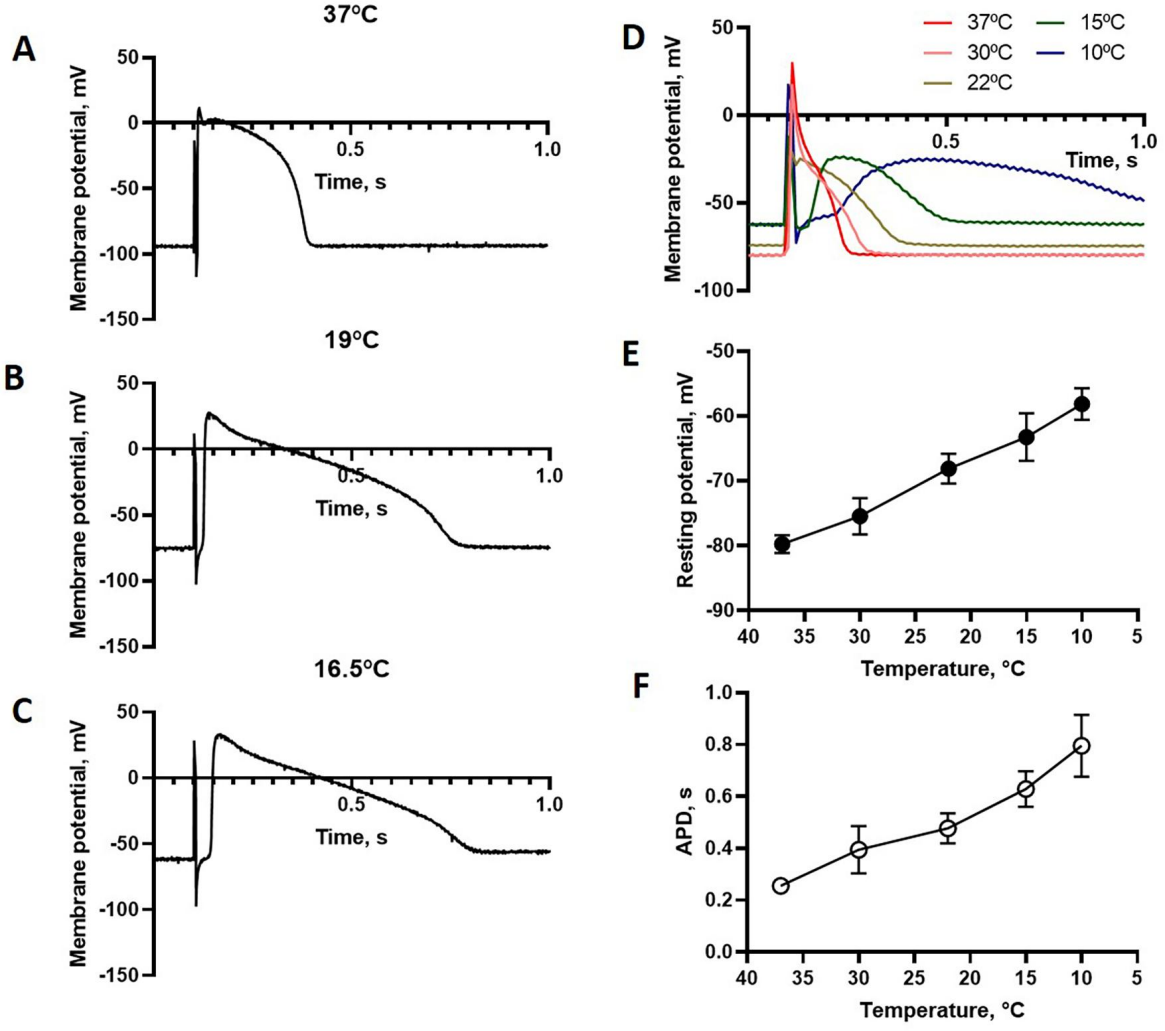
Electrophysiological recordings of membrane potential in isolated cardiac trabeculae at different temperatures. **(A–C)** Shows recordings using point stimulation and **(D)** recordings using field stimulation. **(E,F)** Show summarized data (field stimulation) of resting membrane potential and action potential duration (APD), respectively, *n* = 6.

Our next step was to investigate the electrophysiological and contractile properties in isolated trabecular muscles at different temperatures. Figures 4A–D shows electrophysiological recordings of membrane potential in paced trabecular preparations at different temperatures. Cooling depolarizes the resting membrane potential and prolongs the action potential duration. At the lower temperatures, the responses were dominated by the sustained plateau phase. In these experiments (Figures 4A–C) using point stimulation with bipolar electrodes, the initial stimulation artifacts were minimal, and we observed that the initial fast upstroke phase was attenuated at lower temperature. However, the point stimulation failed to elicit propagated action potentials at temperature below 15°C. We assume that this is due to poor propagation of the action potential within the trabeculae. Consistent with this assumption, a delay between stimulation and action potential upon cooling was observed applying point stimulation (Figures 4A–C). The effects of temperature on propagation are possibly associated with an attenuation of the initial upstroke phase. We therefore used field stimulation of the whole preparation which enables the recording of action potential duration down to 10°C. Using this approach (Figure 4D), the initial phase of the action potential was not distinguishable from the stimulation artifact, but action potential duration could be adequately recorded over the whole temperature range. The resting membrane potential became less polarized (Figure 4E), and the action potential duration (APD) longer (Figure 4F) at lower temperature.

**Figure 5:**
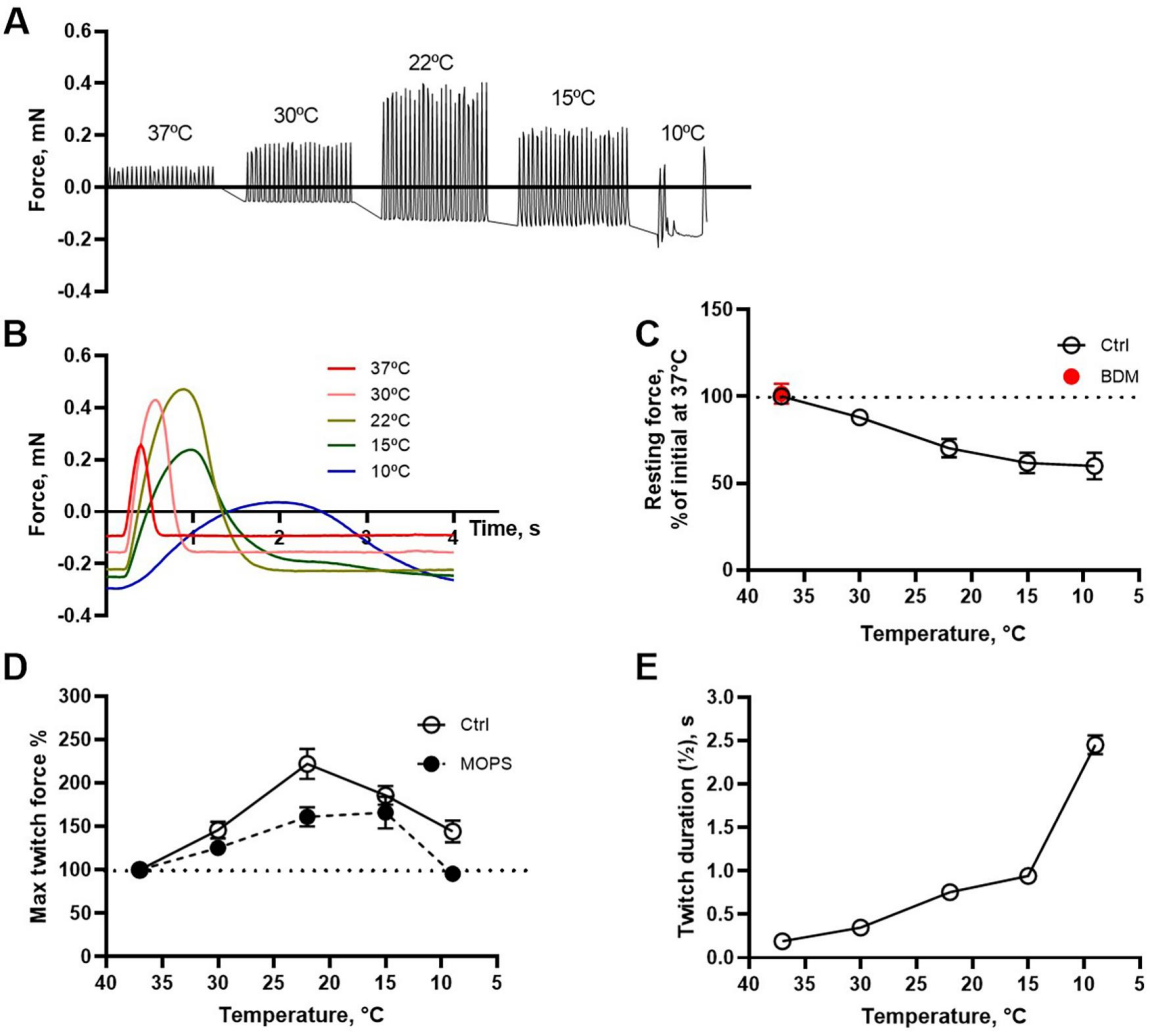
Mechanical recordings of force in isolated intact cardiac trabeculae at different temperatures. **(A,B)** Show original traces of a trabecular preparation stimulated at different temperatures. Note the increased twitch force at 22°C compared to that at 37°C and the gradually decreased resting tension with lower temperature. **(C–E)** Summarize the values of resting tension, maximal twitch force and twitch duration. *N* = 4–11. Twitch force was also evaluated in MOPS-buffered solution **(D)** addition of 50 mM BDM (red symbol, **C**) did not change resting force at 37°C.

Figure 5A shows an original isometric force recording from a paced trabecular preparation. Under these *in vitro* conditions, the developed (twitch) force was maximal at 22°C, similar to the developed pressure measured in the whole hearts (cf. Figure 3B). Figure 5B shows the recordings with higher time resolution. Figures 5C–E summarize the effects of temperature on the resting force, maximal twitch force and the duration of the twitches, showing a prolonged twitch duration at lower temperature and a maximal force at 22°C. The rate of force development (cf. Figure 5B) decreased with lowered temperature. The experiments were performed in carbogen gassed Krebs-Ringer solution where temperature has small effects on pH. We therefore repeated the experiments in MOPS buffered solution (which is not temperature sensitive) and found similar temperature dependence of active force (Figure 5D).

We also observed that the tone in the relaxed state was decreased with lower temperature (Figures 5A,C). The resting force at 37°C was not reduced by 50 mM BDM (Figure 5C), showing that there is no active tone involved in the relaxed state. This suggests that the resting force is affected by temperature not acting via mechanisms involved in active force.

### 3.3 Force and calcium sensitivity of permeabilized preparations

**Figure 6:**
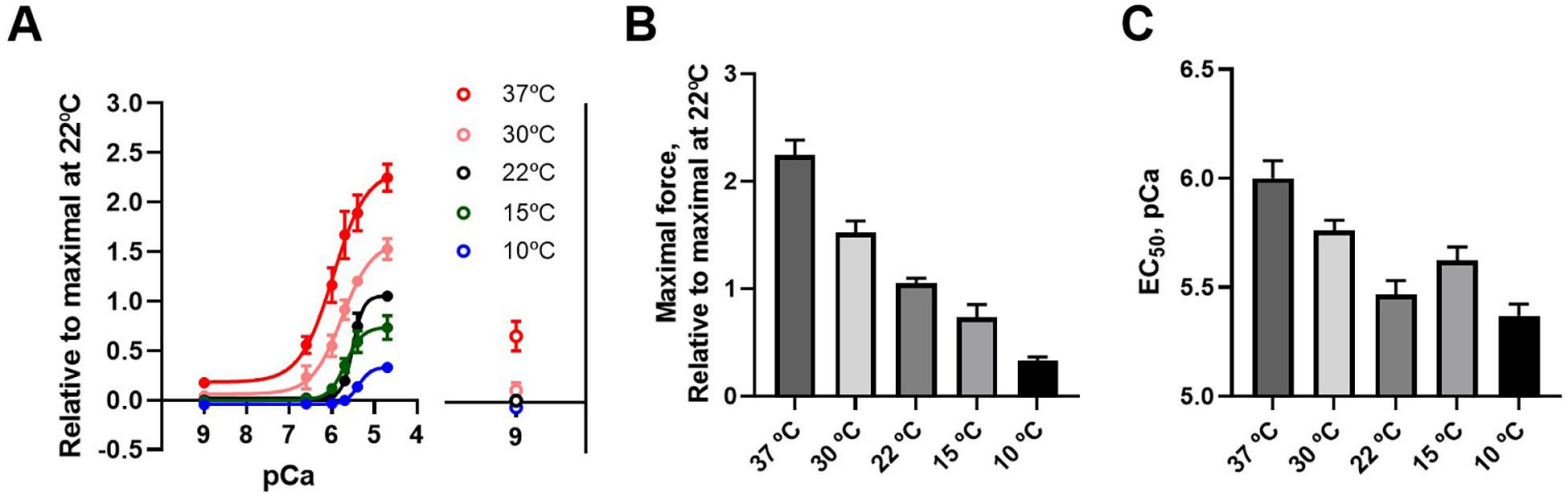
Ca^2+^ sensitivity of force in permeabilized preparations at different temperatures. [Ca^2+^] is given in pCa units and force is normalized to the initial force of contraction at 22°C **(A)** A hyperbolic equation is fitted to each curve. Symbols to the right show the force at pCa 9 after exposure to the [Ca^2+^] steps, *n* = 6 in each group. **(B)** shows the average maximal force and **(C)** shows the EC_50_ values (i.e., [Ca^2+^] giving half of the maximal force).

Using permeabilized preparations, the force generation and calcium sensitivity of the contractile system was determined at different temperatures (Figure 6). For all preparations, an initial contraction at pCa 4.7 was recorded at 22°C and used for normalization. At lower temperature, maximal activated force at saturating calcium concentration decreased, and the calcium sensitivity was reduced (shown by the rightward shift of the curves). Figures 6B,C summarize the data, showing a decreased force and Ca^2+^-sensitivity at lower temperature. The experiments were performed at 5 mM MgATP with 15 mM phosphocreatine and in the presence of creatine kinase. We also performed experiments at 37°C with 10 mM MgATP and 20 mM phosphocreatine showing similar results (i.e., higher tension at 37 compared to 22°C).

### 3.4 Small angle x-ray diffraction

**Figure 7:**
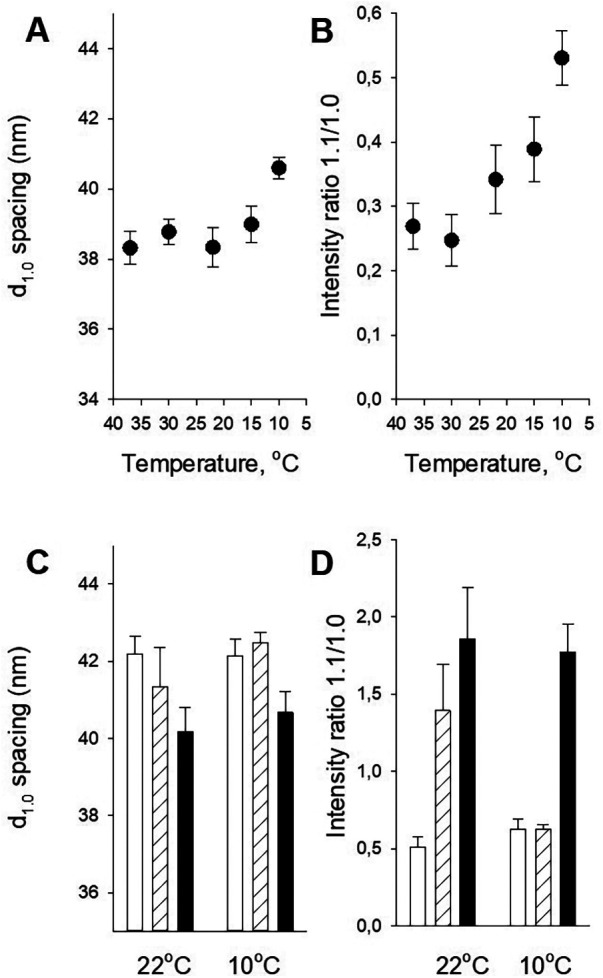
Contractile filament structure determined with small angle x-ray scattering. **(A,B)** Show the temperature dependence of the d_1.0_ spacing and the intensity ratio between 1.1 and 1.0 reflections in intact preparations, respectively, *n* = 3–12. **(C,D)** Show the d_1.0_ spacing and the 1.1/1.0 intensity ratio in permeabilized preparations, *n* = 5–13. Open bars show data in relaxed fibers (pCa 9), cross-hatched bars activated fibers (pCa 4.7) and full bars from fibers in rigor.

To examine the effects of temperature on the organization of the contractile filament structure and the positioning of the myosin heads, we performed small angle x-ray diffraction measurements. The 1.0 and 1.1 equatorial reflections were clearly observed in the pig heart samples c.f (24). and was resolved at all temperatures. In the intact fibers, a swelling of about 6% of the lattice spacing was observed when temperature was decreased below 22°C (Figure 7A). As seen in Figure 7B, lower temperatures also gave an increase in the 1.1/1.0 intensity ratio, suggesting movement of myosin heads towards actin. In contrast to data from intact muscle, we could not observe a significant swelling of the lattice in relaxed permeabilized fibers between 22°C and 10°C (open bars in Figure 7C). A small shrinkage was observed at both temperatures in rigor (black bars in Figure 7C). At 22°C, contraction in pCa 4.7 (hatched bars in Figure 7D) was associated with an increase in the 1.1/1.0 intensity ratio, approaching that in rigor. In contrast, the ratio did not increase significantly following exposure to pCa 4.7 at 10°C.

## 4 Discussion

Variation in temperature is a key physical process influencing cardiac function. Apart from the effects induced by extreme external conditions, cooling and rewarming of the heart are important clinical events, e.g., during cardiac surgery. Still, the effects on the different steps in cardiac excitation and contraction are poorly understood. In particular, data are needed in relation to evaluation of cardiac beating *ex vivo*, prior to transplantation (13, 19). For hearts of larger animals, including the clinically important pig, little is known. We report here on the effects in a broader temperature interval on several cardiac parameters, and demonstrate a biphasic effect on systolic pressure, with an increase during moderate cooling and a decrease in the lower temperature interval.

An obvious effect of lowering temperature is the significant decrease in heart rate, which is a well-known phenomenon influencing the isolated heart e.g., (29), possibly via direct effects on pacemaker channel (HCN) (30). Similarly to previous findings (31), we show that cooling is associated with changes in ECG parameters with a slowing of the atrio-ventricular conduction time (PQ), and prolonged action potential duration (QT). At temperatures below 15°C total AV block occurred in several hearts. At the cellular level, the action potentials are significantly prolonged and below 30°C the fast upstroke phase is attenuated. The latter effect clearly suggests an inhibition of the fast Na^+^ current, possibly via direct temperature effects on the channel, or indirect influence associated with a gradual cellular depolarization. The changes in the upstroke rate will affect the propagation velocity of the excitation as seen in the ECG parameters, and possibly also lead to spatial inhomogeneities underlying the risks for severe ventricular arrhythmias. The effects on the action potential plateau clearly reflect slower activation and deactivation of the L-type calcium channels. The risk for ventricular fibrillation in the cold is evident, and ethanol seems to prevent this (21), which was the reason for including this in the study where we focused on contractility. We did not observe any major effects of ethanol at 37°C, but membrane stabilization of the compound is most likely present in the cold, although further studies are needed. The major effects of cooling on conduction are most likely directly associated with the temperature effect, which is consistent with clinical observations showing AV block in the cold (32, 33).

Interestingly, the developed pressure of whole hearts, and force in isolated trabeculae, are increased when temperature is lowered from the physiological 37°C–22°C. This is not primarily associated with the decrease in heart rate, since the phenomenon is observed also in the isolated paced trabecular muscle. The lowered Ca^2+^ sensitivity at lower temperature in permeabilized trabeculae speak against an increased myofibrillar Ca^2+^ sensitively in the range 37°C–22°C as an explanation for the active pressure and force increase. Rather, increased intracellular Ca^2+^ levels and the longer twitch duration seem to override the lower sensitivity in this temperature range. Increased [Ca^2+^] has been reported at lower temperature (34), and associated with a slower Ca^2+^ removal process (35). At temperatures below room temperature, developed pressure and active force of whole heart and isolated trabeculae drop significantly, which most likely is due to the very low sensitivity to activator Ca^2+^ as shown in the permeabilized fibers from the pig heart. This is similar to effects found in the rabbit heart (36). It is currently unclear if this is due to direct effects on the troponin-tropomyosin system, the cross-bridge reactions controlled by the thin filament system or possibly due to altered troponin phosphorylation (37). The latter possibility seems less likely since the permeabilized preparations, lose most kinases and phosphatase activities.

The low force responses at low temperature (<22°C) seem to be due to an activation problem in the thin filament regulatory system. Consistent with this notion, our x-ray scattering experiments on permeabilized preparations, showed unaltered 1.1/1.0 intensity ratio at 10°C upon Ca^2+^ activation, suggesting that cross-bridges are not approaching the thin filaments, although they are able to attach in rigor. In contrast, in the relaxed intact muscle lowering temperature is associated with a gradual mass transfer of myosin heads (increased 1.1/1.0 intensity ratio) and a swelling of the filament lattice (increased d_1.0_ spacing). These changes in myosin head position in intact relaxed cardiac muscle are not associated with force generation. Other mechanical consequences related with cooling remain to be investigated. The increased filament distances and swelling of the sarcomere unit in the cold, suggest that cold temperature affect the filament structure most likely due to changes in the intracellular environment.

Although clinical cooling in the range 37°C–30°C has been used, our data show that the pig heart can beat down to 22, 15 and even 10°C. It can be noted that the optimal temperature for cardiac contraction can vary between species, and is lower in small rodents (38). For the pig and human hearts, the risks for cardiac arrhythmias in the cold are commented on above and cardiac output would most likely be very low under these conditions due to low frequency and decreased systolic pressure. Whether this is compensated by a lower vascular resistance and lower metabolism and/or affected by altered O_2_ release from hemoglobin, is unclear. According to a case report, a patient with accidental severe hypothermia with body temperature of 13.7°C, asystole and isoelectric EEG, was resuscitated to good physical and mental health. This suggests that body organs including the heart, can survive down to severe hypothermia (3).

In summary, these experiments on the pig heart identify different phases in the responses to cooling. Initially, in the range 37°C–22°C active pressure and force production are significantly increased, most likely due to increased intracellular [Ca^2+^]. At the same time, the initial upstroke phase of the action potential is attenuated, the heart rate decreased and conduction slowed, which can be associated with the significant risks for arrhythmias associated with changes in body temperature (39). Below room temperature, pressure and force production are significantly inhibited, which most likely is due to failure in the activation system. It should also be noted that cardiac cellular physiology is altered with a significant swelling of filament lattice (and cellular) volume, by about 14%, from 22°C to 10°C.

## Data Availability

The original contributions presented in the study are included in the article, further inquiries can be directed to the corresponding author.

## References

[1] Paal P Gordon L Strapazzon G Brodmann Maeder M Putzer G Walpoth B Accidental hypothermia-an update: the content of this review is endorsed by the international commission for mountain emergency medicine (ICAR MEDCOM). Scand J Trauma Resusc Emerg Med. (2016) 24(1):111. 10.1186/s13049-016-0303-7 27633781 PMC5025630

[2] Paal P Pasquier M Darocha T Lechner R Kosinski S Wallner B Accidental hypothermia: 2021 update. Int J Environ Res Public Health. (2022) 19(1):501. 10.3390/ijerph19010501 35010760 PMC8744717

[3] Gilbert M Busund R Skagseth A Nilsen PA Solbo JP. Resuscitation from accidental hypothermia of 13.7 degrees C with circulatory arrest. Lancet. (2000) 355(9201):375–6. 10.1016/S0140-6736(00)01021-7 10665559

[4] Wik L Kiil S. Use of an automatic mechanical chest compression device (LUCAS) as a bridge to establishing cardiopulmonary bypass for a patient with hypothermic cardiac arrest. Resuscitation. (2005) 66(3):391–4. 10.1016/j.resuscitation.2005.03.011 15992987

[5] Bartoli CR Wong R Mazandi VM Fairman AS Maffei FA. Ice water drowning survival after 147-Minute submersion and 7 degrees C hypothermic circulatory arrest. JACC Case Rep. (2025) 30(25):104885. 10.1016/j.jaccas.2025.104885 40883082 PMC12402389

[6] Taccone FS Cariou A Zorzi S Friberg H Jakobsen JC Nordberg P Hypothermia versus normothermia in patients with cardiac arrest and shockable rhythm: a secondary analysis of the TTM-2 study. Crit Care. (2024) 28(1):335. 10.1186/s13054-024-05119-3 39407230 PMC11481803

[7] Tveita T Sieck GC. Physiological impact of hypothermia: the good, the bad, and the ugly. Physiology (Bethesda). (2022) 37(2):69–87. 10.1152/physiol.00025.2021 34632808

[8] Ross DN. Ice arrest of the heart. Lancet. (1961) 2(7197):293–4. 10.1016/S0140-6736(61)90581-5 13743599

[9] Monteagudo Vela M Garcia Saez D Simon AR. Current approaches in retrieval and heart preservation. Ann Cardiothorac Surg. (2018) 7(1):67–74. 10.21037/acs.2018.01.06 29492384 PMC5827116

[10] Steen S Paskevicius A Liao Q Sjoberg T. Safe orthotopic transplantation of hearts harvested 24 h after brain death and preserved for 24 h. Scand Cardiovasc J. (2016) 50(3):193–200. 10.3109/14017431.2016.1154598 26882241 PMC4898163

[11] Qin G Wohlfart B Zuo L Hu J Sjöberg T Steen S. Intact coronary and myocardial functions after 24 h of non-ischemic heart preservation. Scand Cardiovasc J. (2020) 54(1):59–65. 10.1080/14017431.2019.1684553 31692381

[12] Nilsson J Jernryd V Qin G Paskevicius A Metzsch C Sjoberg T A nonrandomized open-label phase 2 trial of nonischemic heart preservation for human heart transplantation. Nat Commun. (2020) 11(1):2976. 10.1038/s41467-020-16782-9 32532991 PMC7293246

[13] Steen S Paskevicius A Liao Q Steen E. *Ex vivo* evaluation of the whole heart function allowing selective investigation of the right and left heart. Scand Cardiovasc J. (2024) 58(1):2418084. 10.1080/14017431.2024.2418084 39460683

[14] Längin M Mayr T Reichart B Michel S Buchholz S Guethoff S Consistent success in life-supporting porcine cardiac xenotransplantation. Nature. (2018) 564(7736):430–3. 10.1038/s41586-018-0765-z 30518863

[15] Längin M Reichart B Steen S Sjöberg T Paskevicius A Liao Q Cold non-ischemic heart preservation with continuous perfusion prevents early graft failure in orthotopic pig-to-baboon xenotransplantation. Xenotransplantation. (2020) 28(1):e12636. 10.1111/xen.12636. 32841431

[16] Reichart B Längin M Radan J Mokelke M Buttgereit I Ying J Pig-to-non-human primate heart transplantation: the final step toward clinical xenotransplantation? J Heart Lung Transplant. (2020) 39(8):751–7. 10.1016/j.healun.2020.05.004 32527674

[17] Griffith BP Goerlich CE Singh AK Rothblatt M Lau CL Shah A Genetically modified porcine-to-human cardiac xenotransplantation. N Engl J Med. (2022) 387(1):35–44. 10.1056/NEJMoa2201422 35731912 PMC10361070

[18] Moazami N Stern JM Khalil K Kim JI Narula N Mangiola M Pig-to-human heart xenotransplantation in two recently deceased human recipients. Nat Med. (2023) 29(8):1989–97. 10.1038/s41591-023-02471-9 37488288

[19] Steen S Liao Q Pascevivius A Li M Steen E. *Ex vivo* resuscitation and evaluation of hearts after 22 min of normothermic cardiac arrest. Scand Cardiovasc J. (2025) 59(1):2525098. 10.1080/14017431.2025.2525098 40553492

[20] Steen S Sjöberg T Liao Q Bozovic G Wohlfart B. Pharmacological normalization of circulation after acute brain death. Acta Anaesthesiol Scand. (2012) 56(8):1006–12. 10.1111/j.1399-6576.2012.02721.x 22651688

[21] Granberg PO. Alcohol and cold. Arctic Med Res. (1991) 50(Suppl 6):43–7. 1811578

[22] Arlock P Li M Davis B Lovdahl C Liao Q Sjoberg T Excitation and contraction of cardiac muscle and coronary arteries of brain-dead pigs. FASEB Bioadv. (2023) 5(2):71–84. 10.1096/fba.2022-00104 36816513 PMC9927844

[23] Lu X Tobacman LS Kawai M. Effects of tropomyosin internal deletion Delta23Tm on isometric tension and the cross-bridge kinetics in bovine myocardium. J Physiol. (2003) 553(Pt 2):457–71. 10.1113/jphysiol.2003.053694 14500764 PMC2343557

[24] Li M Qin Z Steen E Terry A Wang B Wohlfart B Development and prevention of ischemic contracture (“stone heart”) in the pig heart. Front Cardiovasc Med. (2023) 10:1105257. 10.3389/fcvm.2023.1105257 36891241 PMC9986286

[25] Fabiato A. Computer programs for calculating total from specified free or free from specified total ionic concentrations in aqueous solutions containing multiple metals and ligands. Methods Enzymol. (1988) 157:378–417. 10.1016/0076-6879(88)57093-3 3231093

[26] Harrison SM Bers DM. The effect of temperature and ionic strength on the apparent ca-affinity of EGTA and the analogous ca-chelators BAPTA and dibromo-BAPTA. Biochim Biophys Acta. (1987) 925(2):133–43. 10.1016/0304-4165(87)90102-4 3113491

[27] Buffet A Rothkirch A Dohrmann R Korstgens V Abul Kashem MM Perlich J P03, the microfocus and nanofocus x-ray scattering (MiNaXS) beamline of the PETRA III storage ring: the microfocus endstation. J Synchrotron Radiat. (2012) 19(Pt 4):647–53. 10.1107/S0909049512016895 22713902 PMC3380660

[28] Liu J Schwartzkopf M Arner A. Rigor bonds cause reduced sarcomeric volume in skinned porcine skeletal muscle under PSE-like conditions. Meat Sci. (2018) 139:91–6. 10.1016/j.meatsci.2018.01.014 29413682

[29] Black PR van Devanter S Cohn LH. Effects of hypothermia on systemic and organ system metabolism and function. J Surg Res. (1976) 20(1):49–63. 10.1016/0022-4804(76)90083-4 1107674

[30] Wu Y Wang Q Granger J Gaido OR Aguilar EN Ludwig A HCN channels sense temperature and determine heart rate responses to heat. *bioRxiv*. (2023). 10.1038/s41467-025-57358-9 PMC11873294 40025061

[31] Chhabra L Devadoss R Liti B Spodick DH. Electrocardiographic changes in hypothermia: a review. Ther Hypothermia Temp Manag. (2013) 3(2):54–62. 10.1089/ther.2013.0003 24837798

[32] Jacob AI Lichstein E Ulano SD Chadda KD Gupta PK Werner BM. A-V block in accidental hypothermia. J Electrocardiol. (1978) 11(4):399–402. 10.1016/S0022-0736(78)80149-6 712292

[33] Bashour TT Gualberto A Ryan C. Atrioventricular block in accidental hypothermia–a case report. Angiology. (1989) 40(1):63–6. 10.1177/000331978904000112 2910145

[34] Stowe DF Fujita S An J Paulsen RA Varadarajan SG Smart SC. Modulation of myocardial function and [Ca2+] sensitivity by moderate hypothermia in Guinea pig isolated hearts. Am J Physiol. (1999) 277(6):H2321–32. 10.1152/ajpheart.1999.277.6.H2321 10600852

[35] Puglisi JL Bassani RA Bassani JW Amin JN Bers DM. Temperature and relative contributions of ca transport systems in cardiac myocyte relaxation. Am J Physiol. (1996) 270(5 Pt 2):H1772–8. 10.1152/ajpheart.1996.270.5.H1772 8928885

[36] Harrison SM Bers DM. Influence of temperature on the calcium sensitivity of the myofilaments of skinned ventricular muscle from the rabbit. J Gen Physiol. (1989) 93(3):411–28. 10.1085/jgp.93.3.411 2703821 PMC2216215

[37] Schaible N Han YS Hoang T Arteaga G Tveita T Sieck G. Hypothermia/rewarming disrupts excitation-contraction coupling in cardiomyocytes. Am J Physiol Heart Circ Physiol. (2016) 310(11):H1533–40. 10.1152/ajpheart.00840.2015 26993227 PMC4935512

[38] Liu B Wohlfart B Johansson BW. Effects of low temperature on contraction in papillary muscles from rabbit, rat, and hedgehog. Cryobiology. (1990) 27(5):539–46. 10.1016/0011-2240(90)90041-2 2249456

[39] Brown DJ Brugger H Boyd J Paal P. Accidental hypothermia. N Engl J Med. (2012) 367(20):1930–8. 10.1056/NEJMra1114208 23150960

